# Supplementary material for: Gut-Flora-Dependent Metabolite Trimethylamine-N-Oxide Promotes Atherosclerosis-Associated Inflammation Responses by Indirect ROS Stimulation and Signaling Involving AMPK and SIRT1
Source: Nutrients. 2022 Aug 15;14(16):3338. doi: 10.3390/nu14163338 (PMC9416570; doi:10.3390/nu14163338)
Supplement: Supplementary file 1 [file nutrients-14-03338-s001.zip › nutrients-1836533-supplementary.pdf]

Figure S1

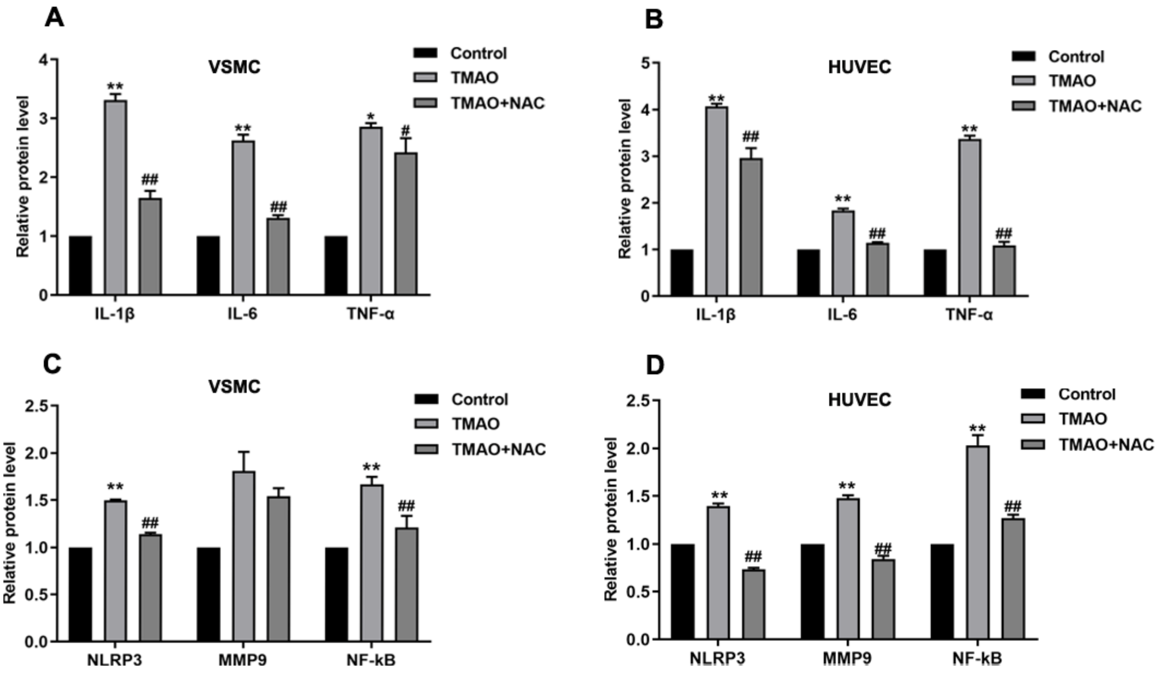

Figure S1.The protein levels of inflammatory cytokines in VSMCs and HUVECs treated with TMAO and NAC. (A, B) Quantification of protein levels of IL-1β，IL-6, TNFα in VSMCs (A) and HUVECs (B) using ImageJ software in Fig.1E and F. (C, D) Quantification of protein levels of NLRP3，MMP9, NF-κB in VSMCs (C) and HUVECs (D) using ImageJ software in Fig.1G and H. Values are mean ± SD; n=3, one-way ANOVA followed by a Tukey post hoc test. *P < 0.05; **P < 0.01 vs. Control; #P < 0.05; ##P < 0.01 vs. TMAO group.

Figure S2

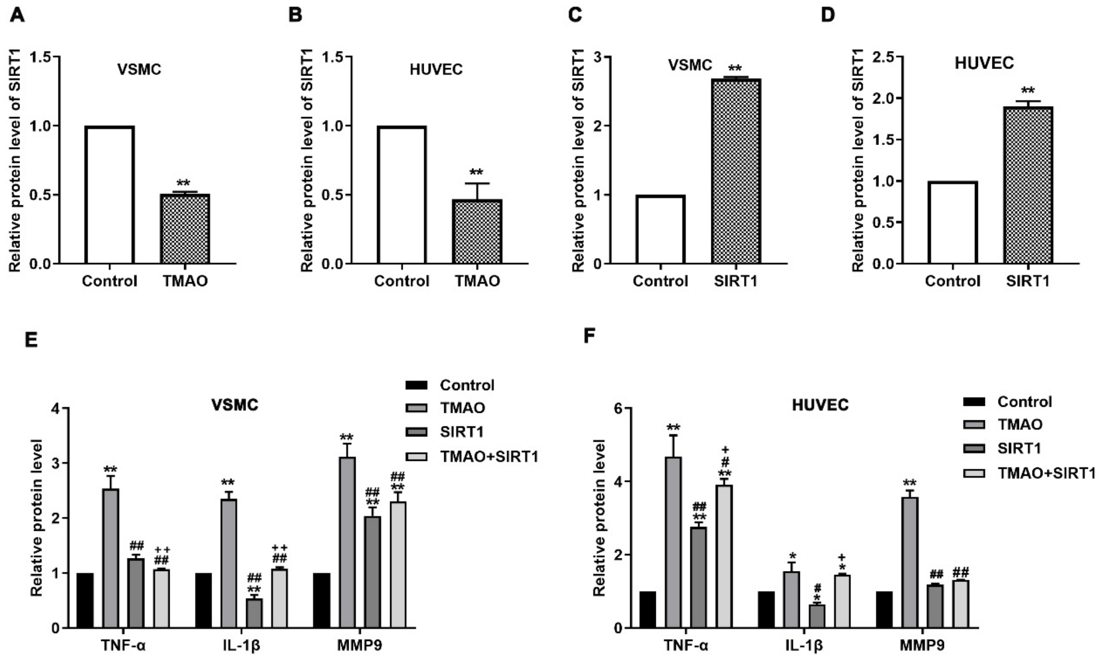

Figure S2. The protein levels of SIRT1 and inflammatory cytokines in VSMCs and HUVECs treated with TMAO, SIRT1 vector and TMAO plus SIRT1 vector. (A, B) Quantification of protein level of SIRT1 after TMAO treatment in VSMCs (A) and HUVECs(B) using ImageJ software in Fig.2A and B. (C, D) Quantification of protein level of SIRT1 in VSMCs (C) and HUVECs (D) transfected with pCMV-Tag 2B-SIRT1 plasmid using ImageJ software in Fig.2C and D. (E, F) Quantification of protein levels of TNFα, IL-1β, MMP9 in VSMCs (E) and HUVECs (F) treated with TMAO, SIRT1 vector and TMAO plus SIRT1 in Fig.2G and H. Values are mean ± SD; n=3, two-way ANOVA followed by a Tukey post hoc test. *P < 0.05; **P < 0.01 vs. Control; #P < 0.05; ##P < 0.01 vs. TMAO group.+ P < 0.05; ++ P < 0.01 vs. SIRT1 group.

Figure S3

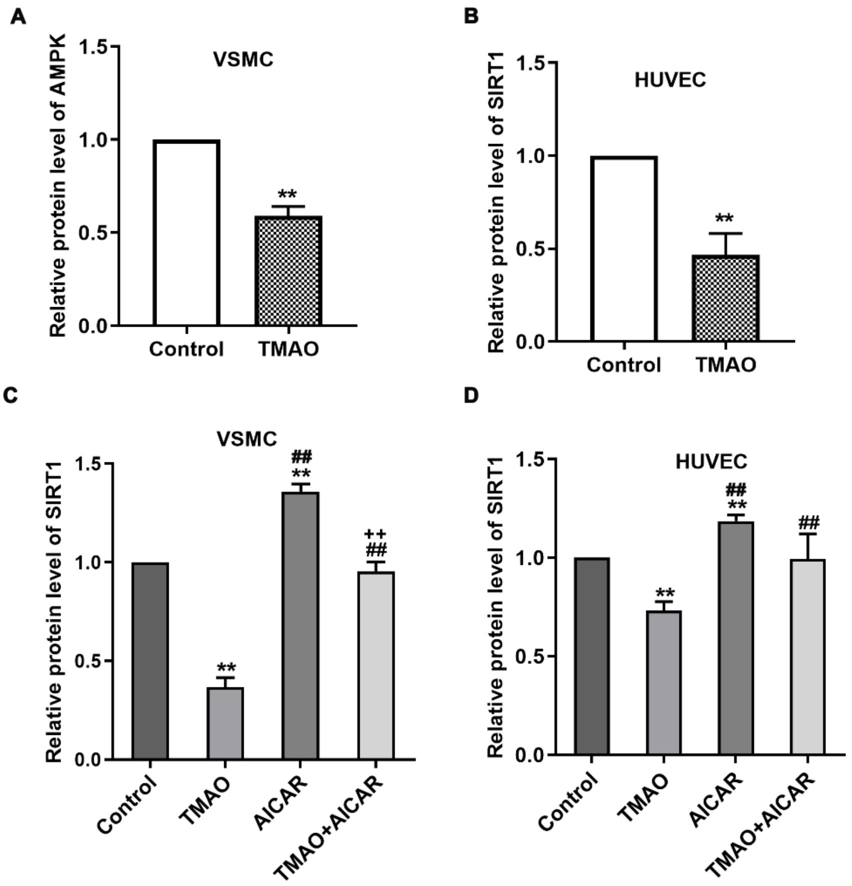

Figure S3. The protein levels of AMPK and SIRT1 in VSMCs and HUVECs treated with TMAO, AICAR, TMAO plus AICAR. (A, B) Quantification of protein level of AMPK after TMAO treatment in VSMCs (A) and HUVECs (B) using ImageJ software in Fig.3A and B. Values are mean ± SD; n=3, Student's *t*-test. **P < 0.01 vs. Control.(C,D) Quantification of protein level of SIRT1 after TMAO, AICAR, TMAO plus AICAR treatment in VSMCs (C) and HUVECs(D) using ImageJ software in Fig.3C and D.Values are mean ± SD; n=3, two-way ANOVA followed by a Tukey post hoc test. **P < 0.01 vs. Control; ##P < 0.01 vs. TMAO group; ++ P < 0.01 vs. AICAR group.

Figure S4

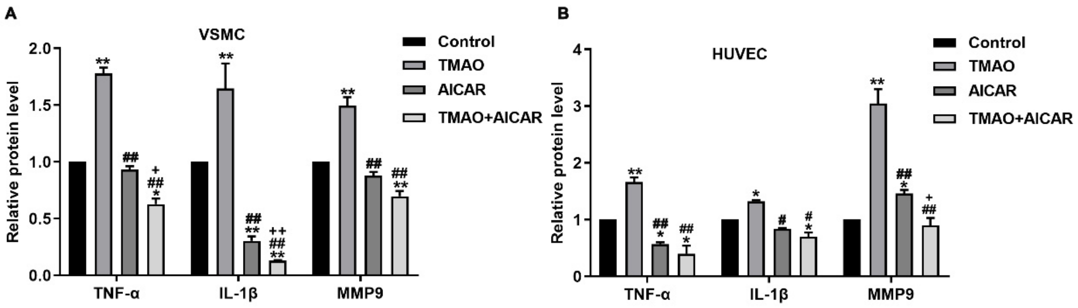

Figure S4. The protein levels of inflammatory cytokines in VSMCs and HUVECs treated with TMAO, AICAR, TMAO plus AICAR. (A,B) Quantification of protein levels of TNFα, IL-1β, MMP9 after TMAO, AICAR, TMAO plus AICAR treatment in VSMCs (A) and HUVECs (B) using ImageJ software in Fig.4C and D. Values are mean ± SD; n=3, two-way ANOVA followed by a Tukey post hoc test. *$P < 0.05$; **$P < 0.01$ vs. Control; #$P < 0.05$; ##$P < 0.01$ vs. TMAO group.+ $P < 0.05$; ++ $P < 0.01$ vs. AICAR group.

Figure S5

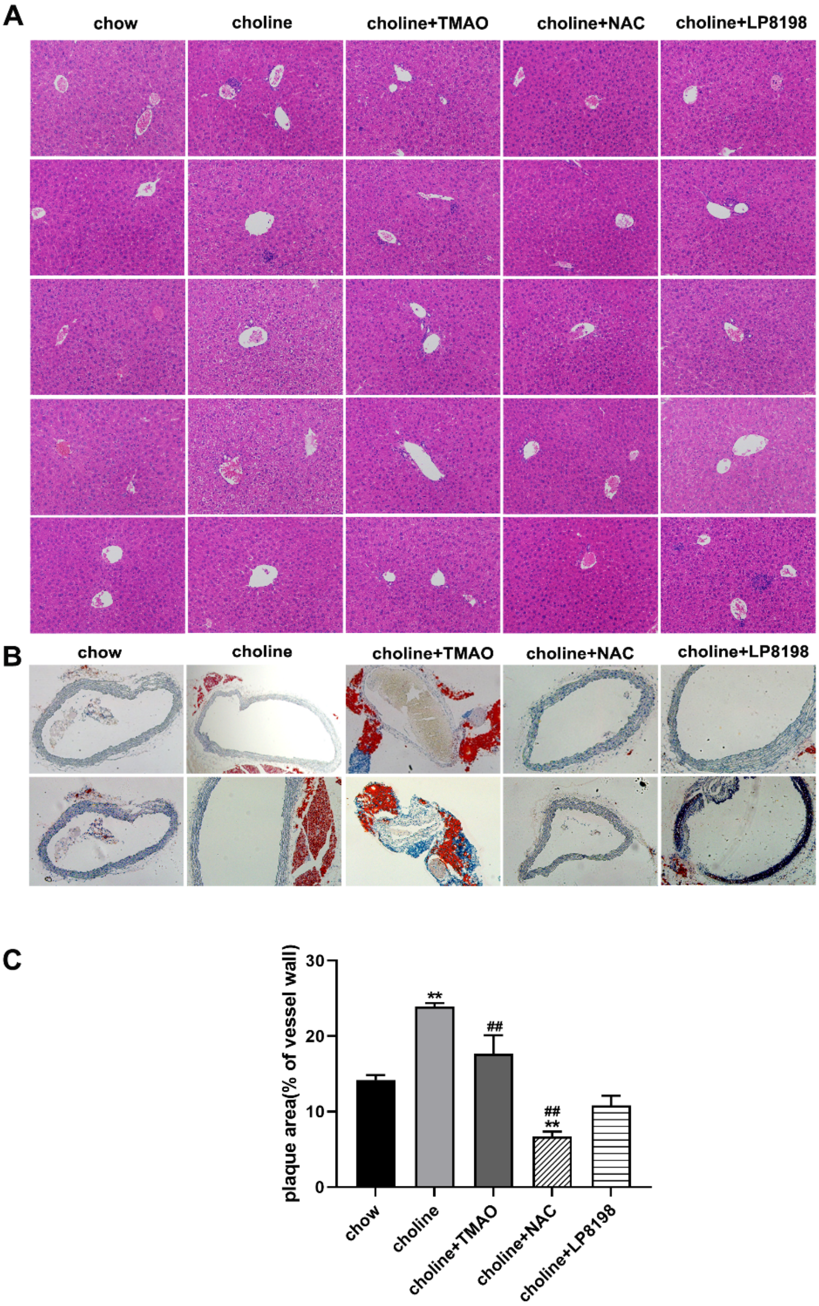

Figure S5. The effect of Choline diet and TMAO on liver and aortic root in C57BL/6 mice. (A) The images of H&E staining at liver in C57BL/6 mice fed with choline diet with or without TMAO, NAC, LP8198 for 12 week. n=5 per group. (B) The images of oil-Red O staining at aortic root in C57BL/6 mice fed with choline diet with or without TMAO, NAC, LP8198 for 12 week. n=2 per group. (C) Statistical analysis of plaque of vessel wall using ImageJ software in (B). n=6 (n=2 per group, each sample was measured three times), two-way ANOVA followed by a Tukey post hoc test. **P < 0.01 vs. chow group; ##P < 0.01 vs. choline group.
